# Machine Learning-Enabled Metallomics Reveals Geographic Exposomic Signatures in a Large Brazilian Cohort

**DOI:** 10.1021/envhealth.6c00155

**Published:** 2026-05-20

**Authors:** Déborah Araújo Morais, Wellington Tavares de Sousa Júnior, Gabriela Pereira de Salles, Marilia Cristina Oliveira Souza, Jose L. Domingo, Paulo Lotufo, Isabela M. Benseñor, Fernando Barbosa

**Affiliations:** † Laboratório de Toxicologia Analítica e de Sistemas (ASTOx). Departamento de Análises Clínicas, Toxicológicas e Bromatológicas, Faculdade de Ciências Farmacêuticas de Ribeirão Preto, University of São Paulo, Ribeirão Preto, SP 14040-903, Brazil; ‡ School of Pharmaceutical Sciences of Ribeirao Preto, Department of Biomolecular Sciences, University of Sao Paulo, Av. do Café s/n, Ribeirao Preto, Sao Paulo 14040-903, Brazil; § Laboratory of Toxicology and Environmental Health, School of Medicine, Universitat Rovira i Virgili, Sant Llorenç 21, Reus, Catalonia 43201, Spain; ∥ Centro de Pesquisa Clínica e Epidemiológica, Hospital Universitário, University of São Paulo, São Paulo, SP 05508-220, Brazil

**Keywords:** metallomics, exposomics, machine learning, geographic exposomic signatures (GExOS), environmental exposure, human biomonitoring

## Abstract

Human exposure to environmental metals and metalloids is shaped by complex interactions among geography, sociodemographic characteristics, lifestyle, and environmental conditions. Although human biomonitoring provides a powerful framework to assess internal exposure, large-scale studies capable of resolving geographically structured exposomic patterns remain scarce. This study aimed to evaluate whether metallomics profiles combined with machine learning can be used to identify Geographic ExposOmic Signatures (GExOS), defined as geographically structured internal exposure patterns derived from integrated biomonitoring data. We profiled the plasma metallome of 9949 adults participating in the Brazilian Longitudinal Study of Adult Health (ELSA-Brasil). Plasma concentrations of 15 metals and metalloids were quantified ICP-MS. Unsupervised multivariate analyses were used to explore global metallomics patterns, followed by supervised machine learning models (XGBoost) to assess geographic classification performance and identify the elements driving regional differentiation. Distinct plasma metallomics profiles were observed according to sex, age, education, income, smoking, and alcohol consumption. Unsupervised analyses revealed structured but continuous regional exposure gradients. Machine learning models demonstrated robust geographic classification performance, with accuracy, sensitivity, and specificity consistently exceeding 80%. Rubidium emerged as a major determinant of geographic discrimination, highlighting its role in shaping spatially structured internal exposure patterns and defining GExOS. These findings demonstrate that the integration of metallomics and machine learning enables the identification of GExOS, providing a scalable and biologically grounded framework to resolve spatial heterogeneity in the human exposome. Metallomics-derived GExOS encode interpretable and predictive exposure signatures with potential applications in environmental surveillance, population health risk assessment, exposure mapping, nutritional epidemiology, and forensic and regulatory sciences.

## Graphical abstract

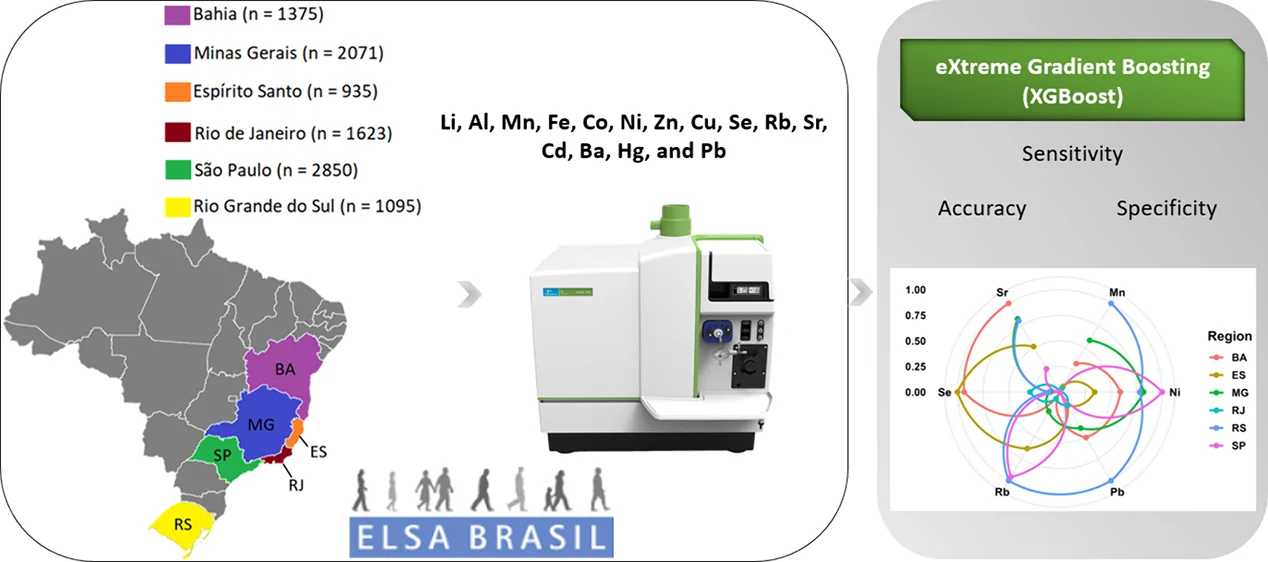

## 1 Introduction

Omics sciences, including genomics, proteomics, metabolomics, and metallomics, have become central to modern biomedical and environmental research, enabling comprehensive characterization of biological systems across multiple molecular layers.^1,2^ Among these approaches, metallomics focuses on the systematic study of metal and metalloid species in biological matrices and their interactions with biomolecules, metabolic pathways, and physiological processes. Because metals play dual roles as essential nutrients and environmental toxicants, metallomics has gained relevance in toxicology, food and nutritional sciences, public health, and environmental health research,^3,4^ as well as in forensic science, where elemental signatures are increasingly used to support source attribution and individual profiling.^5^

In parallel, the rapid evolution of multiomic platforms and computational data integration strategies has transformed the way complex exposure–health relationships are investigated. The integration of omics data through data mining and machine learning approaches allows researchers to move beyond single-exposure analyses toward the identification of multidimensional biological patterns and biomarkers associated with health outcomes. By applying advanced algorithms capable of handling high-dimensional data, studies have demonstrated improved classification of disease states, enhanced understanding of underlying mechanisms, and new opportunities for targeted prevention and intervention strategies.^6–9^

Within this framework, metallomics offers a unique window into the human exposome, as plasma elemental profiles integrate environmental exposures, dietary intake, lifestyle factors, and physiological regulation.^10^ Because metals and metalloids reflect both external exposures and internal biological processes, their integrated measurement provides a biologically meaningful representation of the internal chemical environment. Geographic, social, and environmental heterogeneity can therefore be reflected in internal metal distributions, generating structured exposure signatures that vary across populations and regions.^11,12^

Emerging evidence supports the relevance of metallomic approaches for exposome research, including their potential to uncover disease-related exposure patterns, resolve geographic variability in environmental exposures, and identify biologically relevant exposure signatures in human populations.^10–12^ However, despite this recognized potential, few large-scale population-based studies have explored the capacity of metallomics to resolve spatially structured internal exposure patterns, particularly in low- and middle-income countries, where environmental heterogeneity and exposure disparities are often substantial.^13^

Recent advances in high-dimensional biomonitoring and computational analysis provide new opportunities to operationalize the spatial dimension of the exposome. In particular, the integration of metallomics with machine learning enables the identification of complex, multidimensional exposure patterns that are not discernible using conventional statistical approaches. Machine learning algorithms can detect latent structures within high-dimensional omics data sets, allowing the identification of reproducible exposure signatures reflecting the combined influence of environmental, dietary, and sociodemographic determinants.^10,14,15^

To formalize this conceptual and analytical framework, we introduce the concept of Geographic ExposOmic Signatures (GExOS) defined as geographically structured internal exposure patterns derived from integrated omics-based biomonitoring data. Within this context, metallomics provides a quantitative and biologically grounded representation of the internal exposome, while machine learning enables the detection and classification of region-specific exposure signatures embedded within high-dimensional elemental profiles. GExOS represent an operational framework to resolve spatial heterogeneity in the human exposome and to link internal chemical profiles with geographic, environmental, and sociodemographic determinants. This concept extends beyond conventional biomonitoring by transforming elemental concentration data into integrative, data-driven exposure signatures with potential applications in environmental epidemiology, risk assessment, precision public health, and forensic science.

Then, in this study, we applied a metallomics-based strategy combined with supervised machine learning to identify Geographic ExposOmic Signatures within a large and geographically diverse population. Using baseline data from the Brazilian Longitudinal Study of Adult Health (ELSA-Brasil), we analyzed plasma concentrations of 15 metals and metalloids, aluminum (Al), barium (Ba), cadmium (Cd), cobalt (Co), copper (Cu), iron (Fe), lithium (Li), manganese (Mn), mercury (Hg), nickel (Ni), lead (Pb), rubidium (Rb), selenium (Se), strontium (Sr), and zinc (Zn), in nearly 10,000 adults. The eXtreme Gradient Boosting (XGBoost) algorithm was employed to classify individuals according to their Brazilian state of residence (Bahia, Minas Gerais, Espírito Santo, Rio de Janeiro, São Paulo, and Rio Grande do Sul). By integrating plasma metallomic profiles with machine learning, this approach enables the identification and characterization of GExOS, providing novel insights into how environmental contamination, dietary patterns, and sociodemographic factors shape internal chemical exposures across geographically distinct populations.

## 2 Methods

### 2.1 Design and Study Population

The Brazilian Longitudinal Study of Adult Health (ELSA-Brasil) is a large prospective cohort composed of 15,105 civil servants recruited from six major public teaching and research institutions across Brazil. These include the Federal Universities of Bahia (UFBA), Espírito Santo (UFES), Minas Gerais (UFMG), and Rio Grande do Sul (UFRGS), as well as the University of São Paulo (USP) and the Oswaldo Cruz Foundation (FIOCRUZ) in Rio de Janeiro. Detailed descriptions of the cohort design, recruitment procedures, and participant characteristics have been published elsewhere.^16–18^

The present investigation is based on a cross-sectional evaluation of baseline data collected between 2008 and 2010 within the ELSA-Brasil cohort. Plasma samples from 9949 participants were analyzed, representing all six study centers located in the Brazilian state capitals of Salvador, Rio de Janeiro, Vitória, Belo Horizonte, São Paulo, and Porto Alegre, corresponding to the states of Bahia, Rio de Janeiro, Espírito Santo, Minas Gerais, São Paulo, and Rio Grande do Sul, respectively. Among these sites, Salvador, Rio de Janeiro, and Vitória are situated in coastal regions, whereas the remaining centers are located inland.

The study was conducted in accordance with the ethical principles outlined in the Declaration of Helsinki. The research protocol was approved by the Institutional Research Ethics Committee of the Faculdade de Ciências Farmacêuticas de Ribeirão Preto, University of São Paulo, Ribeirão Preto, SP, Brazil (CAAE number: 67616023.6.0000.5403), and all participants provided written informed consent.

### 2.2 Other Baseline Covariates

Sociodemographic data was obtained through standardized questionnaires administered at baseline. Information collected included participant age (in years), sex, level of education (categorized as less than high school, high school or some college, and completed college or higher), self-declared race/ethnicity (White, Mixed, Black, Asian, or Indigenous), average monthly household income expressed in U.S. dollars (<1245, 1245–3319, or ≥3320; exchange rate US$1 = R$2), per capita income, and smoking and alcohol consumption status (never, former, or current).

Leisure-time and commuting physical activity were evaluated using the long version of the International Physical Activity Questionnaire (IPAQ). Based on established scoring guidelines, participants were classified into three categories: inactive, insufficiently active, or active.^19^

Body mass index (BMI) was calculated by dividing measured body weight by the square of height (kg/m^2^). BMI was examined both as a continuous variable and as a categorical variable. According to World Health Organization criteria,^20^ participants were classified as underweight (<18.5 kg/m^2^), normal weight (18.5–24.9 kg/m^2^), overweight (25.0–29.9 kg/m^2^), or obese (≥30.0 kg/m^2^).

Clinical conditions were defined using standardized criteria. Hypertension was considered present in participants reporting antihypertensive medication use or presenting measured systolic blood pressure ≥ 140 mmHg and/or diastolic blood pressure ≥ 90 mmHg at examination. Dyslipidemia was defined as LDL-cholesterol levels above 3.37 mmol/L or current use of lipid-lowering medication. Diabetes mellitus was identified based on a prior medical diagnosis, use of glucose-lowering therapy, fasting plasma glucose ≥ 126 mg/dL, HbA1c ≥ 6.5%, or a 2-h oral glucose tolerance test ≥ 200 mg/dL.

### 2.3 Sample Collection

Venous blood samples were collected after overnight fasting into EDTA tubes. Plasma was obtained by centrifugation at 3000 rpm for 15 min, transported to the laboratory at room temperature, and stored at −80 °C until the day of analysis.

### 2.4 Plasma Metallomic Analysis

Plasma concentrations of 15 elements (Al, Ba, Cd, Co, Cu, Fe, Pb, Li, Mn, Hg, Ni, Rb, Se, Sr, and Zn) were quantified using inductively coupled plasma mass spectrometry (ICP-MS) equipped with a quadrupole ion deflector (NexION 2000, PerkinElmer, Shelton, CT, USA). Prior to instrumental analysis, plasma aliquots were prepared by dilution at a ratio of 1:20 using an acidic surfactant solution composed of 0.5% (v/v) nitric acid and 0.005% (v/v) Triton X-100 and Rh as internal standard (10 μg/L), according to an established analytical protocol (Batista et al., 2009). The ICP-MS was calibrated with matrix-matched calibration standards. For matrix-matching purposes, we used a base pool of plasma derived from undosed animals (goats).

All procedures were conducted under strict clean conditions. Sample preparation and analyses were performed in a Class 1000 cleanroom using high-purity reagents. Solutions were prepared with ultrapure deionized water (18.2 MΩ·cm) obtained from a Milli-Q system (Millipore, Bedford, MA) following reverse osmosis purification. Nitric acid (HNO_3_) was further purified by sub-boiling distillation in a quartz system (Kürner Analysentechnik). All laboratory materials, including tubes and containers, were acid-washed and precleaned with nitric acid prior to use. Procedural blanks were analyzed daily to monitor potential contamination throughout the analytical process.

Analytical performance was evaluated through the analysis of a reference material (QM-S-Q1907) provided by the Institut National de Santé Publique du Québec (INSP, Canada). Measured concentrations agreed with target values (*t*-test, 95%). For trace elements not included in the reference material, recovery and precision were evaluated using spiked plasma samples. Concentrations below the corresponding limits of detection (LOD) were handled by imputation using LOD divided by the square root of two (LOD/√2).

Detailed analytical quality assurance and quality control (QA/QC) parameters for each element are presented in Supporting Information (Table S1). To minimize potential batch effects, study samples were randomized across analytical runs, and quality control samples were analyzed after every 30 study samples to monitor instrumental performance. Instrumental drift and signal fluctuations were primarily controlled through internal standardization (Rh) and additional QC-based evaluation procedures were applied to verify analytical stability throughout the analytical sequence.

### 2.5 Statistical Analysis

Categorical variables at baseline (BMI, education level, race, family net income strata, physical activity, smoking habits, alcohol consumption, and clinical conditions, such as hypertension, dyslipidemia, and diabetes) were expressed as absolute numbers (percentages) and compared using the Chi-square test. The Kolmogorov–Smirnov test was applied to assess the normality of data distribution. Age and plasma levels of metals and metalloids (μg/L) were expressed as medians and interquartile ranges (IQR). Differences between Brazilian states were evaluated using the Kruskal–Wallis test, followed by Bonferroni’s post hoc correction for multiple comparisons.

Unsupervised dimensionality reduction was conducted using a combined principal component analysis (PCA) and uniform manifold approximation and projection (UMAP) framework to visualize low-dimensional patterns in plasma metallomic profiles across Brazilian states. Metal concentrations were standardized using z-score normalization (mean-centered and scaled to unit variance). PCA was initially applied for dimensionality reduction, while principal components explaining at least 80% of the cumulative variance were retained. These components were subsequently projected into two dimensions using UMAP to facilitate visualization of regional clustering patterns.

To further investigate multimetal patterns across regions, standardized metal concentrations were analyzed using hierarchical clustering. Results were visualized through a heatmap to identify region-specific metallomic fingerprints, emphasizing differences in exposure profiles driven by combinations of metals rather than isolated elements.

The eXtreme Gradient Boosting (XGBoost) machine learning algorithm was implemented to classify participants according to their state of residence (Bahia, Minas Gerais, Espírito Santo, Rio de Janeiro, São Paulo and Rio Grande do Sul) based on the plasma concentrations of the analyzed metals and metalloids: Li, Al, Mn, Fe, Co, Ni, Zn, Cu, Se, Rb, Sr, Cd, Ba, Hg, and Pb. Model performance was evaluated using accuracy, sensitivity, and specificity derived from the confusion matrix. Accuracy was defined as the proportion of correctly classified instances among all samples. Sensitivity and specificity were calculated using a one-vs-rest approach for each class, where sensitivity represents the proportion of correctly identified samples from a given region, and specificity represents the proportion of correctly identified samples not belonging to that region. The reported values correspond to the average performance across all classes. All statistical analyses were conducted in R software (version 4.2.1), and statistical significance was defined at *p* ≤ 0.05.

## 3 Results and Discussion

### 3.1 Characteristics of the Study Population

The socio-demographic and clinical characteristics of the study population, stratified by State, are summarized in Table 1. Participants from Rio de Janeiro exhibited the lowest median age (49 years), whereas those from Rio Grande do Sul showed the highest (55 years) compared to other regions.

**Table 1:** Sociodemographic and General Characteristics of the Population Described by States, ELSA-Brasil baseline (2008-2010; *n* = 9949)

	Bahia (*n* = 1375)	Minas Gerais (*n* = 2071)	Espírito Santo (*n* = 935)	Rio de Janeiro (*n* = 1623)	São Paulo (*n* = 2850)	Rio Grande do Sul (*n* = 1095)	
	*n* (%)	*n* (%)	*n* (%)	*n* (%)	*n* (%)	*n* (%)	*p*-value
age, median (IQR^a^)							
years	53 (47 – 60)	52 (46 – 59)	52 (46 – 59)	49 (44 – 54.5)	52 (46 – 58)	55 (46 – 62)	<0.001
BMI							
underweight	16 (1.16)	28 (1.35)	9 (0.96)	7 (0.43)	23 (0.81)	7 (0.64)	0.004
eutrophic	533 (38.79)	799 (38.60)	366 (39.14)	570 (35.12)	1011 (35.47)	376 (34.34)	
overweight	531 (38.65)	827 (39.95)	355 (37.97)	666 (41.04)	1131 (39.68)	464 (42.37)	
obese	294 (21.40)	416 (20.10)	205 (21.93)	380 (23.41)	685 (24.04)	248 (22.65)	
level of formal education							
less than high-school	205 (14.91)	221 (10.67)	147 (15.72)	43 (2.65)	508 (17.83)	165 (15.07)	<0.001
high-school and incomplete college	788 (57.31)	1020 (49.25)	397 (42.46)	682 (42.02)	1606 (56.35)	490 (44.75)	
complete college or more	382 (27.78)	830 (40.08)	391 (41.82)	898 (55.33)	736 (25.82)	440 (40.18)	
race							
Black	455 (33.68)	297 (14.51)	118 (12.67)	223 (13.81)	399 (14.19)	141 (12.97)	<0.001
Mixed	618 (45.74)	737 (36.01)	381 (40.92)	501 (31.02)	602 (21.41)	92 (8.46)	
White	248 (18.36)	954 (46.60)	406 (43.61)	846 (52.38)	1653 (58.78)	829 (76.27)	
Asian	12 (0.89)	43 (2.10)	17 (1.83)	32 (1.98)	125 (4.45)	6 (0.55)	
Indigenous	18 (1.33)	16 (0.78)	9 (0.97)	13 (0.81)	33 (1.17)	19 (1.75)	
family net income (US$)							
≤1245	491 (35.94)	566 (27.42)	295 (31.69)	118 (7.28)	874 (30.83)	269 (24.61)	<0.001
1246 – 3319	642 (47.00)	925 (44.82)	426 (45.76)	787 (48.55)	1213 (42.79)	447 (40.90)	
≥3320	233 (17.06)	573 (27.76)	210 (22.55)	716 (44.17)	748 (26.38)	377 (34.49)	
physical activity							
inactive	231 (16.81)	293 (14.22)	303 (32.51)	181 (11.33)	481 (17.34)	134 (12.30)	<0.001
insufficiently active	448 (32.61)	561 (27.23)	204 (21.89)	447 (27.97)	908 (32.73)	328 (30.12)	
active	695 (50.58)	1206 (58.55)	425 (45.60)	970 (60.70)	1385 (49.93)	627 (57.58)	
smoking							
never	911 (66.30)	1205 (58.18)	548 (58.61)	942 (58.04)	1503 (52.74)	607 (55.43)	<0.001
past	359 (26.13)	620 (29.94)	286 (30.59)	471 (29.02)	887 (31.12)	340 (31.05)	
current	104 (7.57)	246 (11.88)	101 (10.80)	210 (12.94)	460 (16.14)	148 (13.52)	
alcohol intake							
never	169 (12.34)	170 (8.22)	200 (21.48)	156 (9.62)	357 (12.53)	77 (7.04)	<0.001
past	309 (22.55)	370 (17.89)	203 (21.81)	319 (19.67)	612 (21.47)	234 (21.39)	
current	892 (65.11)	1528 (73.89)	528 (56.71)	1147 (70.71)	1881 (66.00)	783 (71.57)	
hypertension							
no	760 (55.39)	1319 (63.69)	590 (63.10)	1066 (65.72)	1866 (65.52)	670 (61.41)	<0.001
yes	612 (44.61)	752 (36.31)	345 (36.90)	556 (34.28)	982 (34.48)	421 (38.59)	
dyslipidemia							
no	648 (47.13)	1170 (56.49)	488 (52.30)	936 (57.67)	1588 (55.72)	539 (49.32)	<0.001
yes	727 (52.87)	901 (43.51)	446 (47.70)	687 (42.33)	1262 (44.28)	555 (50.68)	
diabetes							
no	1124 (81.75)	1777 (85.80)	762 (81.50)	1384 (85.27)	2363 (82.94)	921 (84.26)	0.002
yes	251 (18.25)	294 (14.20)	173 (18.50)	239 (14.73)	486 (17.06)	172 (15.74)	

a IQR: interquartile range.

Educational attainment varied significantly across the cohorts. Most participants from Rio de Janeiro had completed a college degree or higher (55.33%), the highest educational level in the study. Conversely, most volunteers from Bahia reported completing high school or having an incomplete college education (57.31%).

Regarding self-reported race, most participants from Bahia identified as Mixed (45.74%) or Black (33.68%), whereas 76.27% of volunteers from Rio Grande do Sul identified as White. Notably, Salvador, the capital of Bahia, has the highest proportion of Black individuals outside the African continent.^21^

Economic data indicated that most participants had an average family net income between US$1246 and US$3319. However, Rio de Janeiro stood out, with 44.17% of participants reporting income exceeding US$3320.

Regional disparities were also observed in lifestyle factors. Specifically, Rio de Janeiro had the most active population (60.70% physical activity), while unhealthy habits were more prominent in other states. São Paulo recorded the highest smoking prevalence (16%), and Minas Gerais showed the highest rate of alcohol consumption (73%).

At baseline, Bahia had the highest prevalence of diagnosed hypertension (44.61%) and dyslipidemia (52.87%). Diabetes diagnosis rates were comparable between Bahia (18.25%) and Espírito Santo (18.50%).

### 3.2 Unsupervised Identification of Regional Metallomic Patterns

Unsupervised dimensionality reduction using a PCA–UMAP approach revealed structured metallomic patterns across Brazilian regions, although with partial overlap among populations (Figure 1). This partial separation suggests both shared exposure backgrounds and region-specific metallomic patterns, which is consistent with continuous gradients of human exposure rather than discrete regional clusters. Overall, these findings indicate that regional differences in internal metal exposure are multidimensional and cannot be fully captured by single-metal distributions considered in isolation, supporting the presence of structured but nondiscrete population-level exposure signatures.

**Figure 1:**
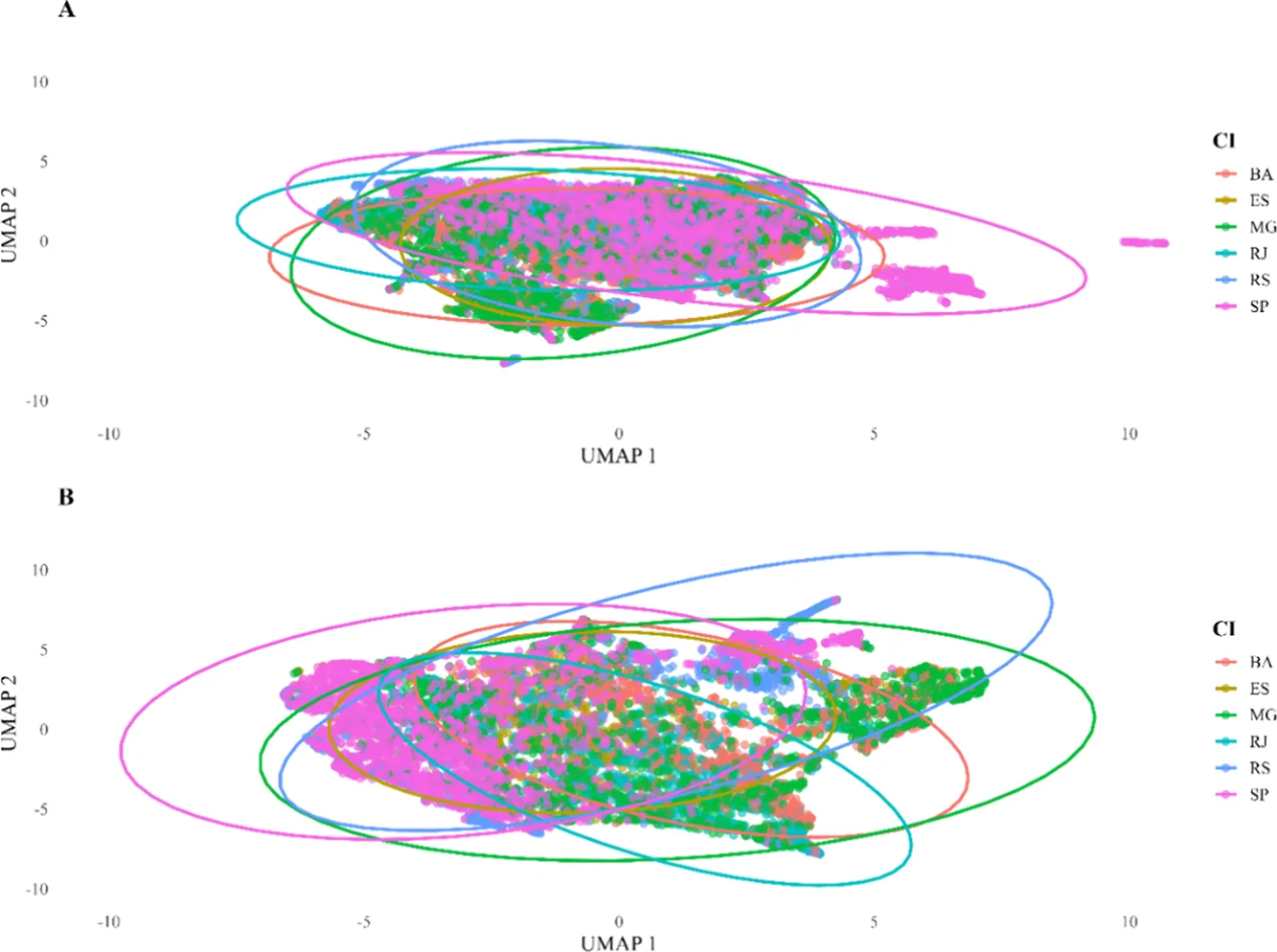
Low-dimensional visualization of human metallomic profiles using a PCA–UMAP approach. Plasma concentrations of all elements (A), and plasma concentrations of Rb, Ni, Se, Ba, Mn, Sr, and Zn (B), were standardized using z-score normalization (mean-centered and scaled to unit variance) and subsequently reduced by principal component analysis (PCA). Principal components explaining at least 80% of cumulative variance were retained and projected into two dimensions using uniform manifold approximation and projection (UMAP). Each point represents one individual, colored according to geographic region (BA, ES, MG, RJ, RS, and SP). Ellipses indicate the 95% data dispersion region for each group.

### 3.3 Region-Specific Metallomic Fingerprints Revealed by Hierarchical Clustering

The plasma distribution of the 15 analyzed metals and metalloids across the Brazilian states is detailed in Supporting Information (Table S2). Participants from Bahia exhibited significantly higher median plasma levels of Co, Cu, and Sr, but lower concentrations of Fe and Rb. In contrast, individuals from Minas Gerais showed elevated levels of Al, Fe, and Zn, but lower concentrations of Hg.

These findings align with regional economic activities. The mining industry is a cornerstone of the Brazilian economy, particularly in Minas Gerais, which accounted for 25% of the country’s mining revenue in 2010. Intense extraction of Fe, Al, and Zn in this region^22^ likely contributes to the higher systemic levels observed in the local population.

In São Paulo, participants showed higher median levels of Ni and Hg, alongside the lowest concentrations of Li, Mn, Se, Cd, Ba, and Pb. The elevated mercury (Hg) levels may be linked to dietary habits. In a previous study^23^ we identified higher Hg concentrations among ELSA-Brasil participants self-identifying as Asian, potentially due to increased seafood consumption. Although only 4.45% of our São Paulo cohort identified as Asian (Table 1), the state hosts the largest Japanese community outside of Japan.^24^ The profound influence of Japanese cuisine in the São Paulo metropolitan area likely promotes higher seafood intake across a broader segment of the population, reflecting the observed Hg profiles.

Participants from Espírito Santo displayed the highest plasma Se concentrations. This is supported by recent evidence that cocoa beans from Espírito Santo have higher Se content than those from Pará, Amazonas, or Bahia,^25^ suggesting a localized dietary source of this micronutrient.

Finally, Rio Grande do Sul exhibited the highest levels of Li, Mn, Rb, Cd, Ba, and Pb, as well as the lowest concentrations of Al, Zn, and Cu. The elevated Pb levels are particularly noteworthy. The state hosts significant Pb mining activities in Caçapava do Sul and one of its largest battery manufacturing plants in Cachoeirinha, both located near the Porto Alegre metropolitan area.^22^

Furthermore, Rio Grande do Sul is Brazil’s leading tobacco-exporting state,^26^ where tobacco cultivation frequently involves the application of Mancozeb, a fungicide associated with increased Mn exposure.^13,27^ Although the ELSA-Brasil population is not occupationally engaged in agricultural work, environmental contamination arising from the widespread use of Mancozeb may still constitute an indirect exposure pathway. Collectively, these industrial, mining, and agricultural activities may represent important environmental contributors to the lead and manganese burden observed among participants from this region.

By integrating metallomics and machine learning, this study provides empirical support for the concept of GExOS, demonstrating that population-level exposure profiles exhibit reproducible and geographically structured patterns.

To further characterize the multimetal combinations underlying the patterns observed in the unsupervised analysis, standardized metal concentrations were examined using hierarchical clustering. The resulting heatmap revealed distinct region-specific metallomic fingerprints driven by combinations of key metals rather than isolated elements, highlighting differences in exposure profiles across populations (Figure 2).

**Figure 2:**
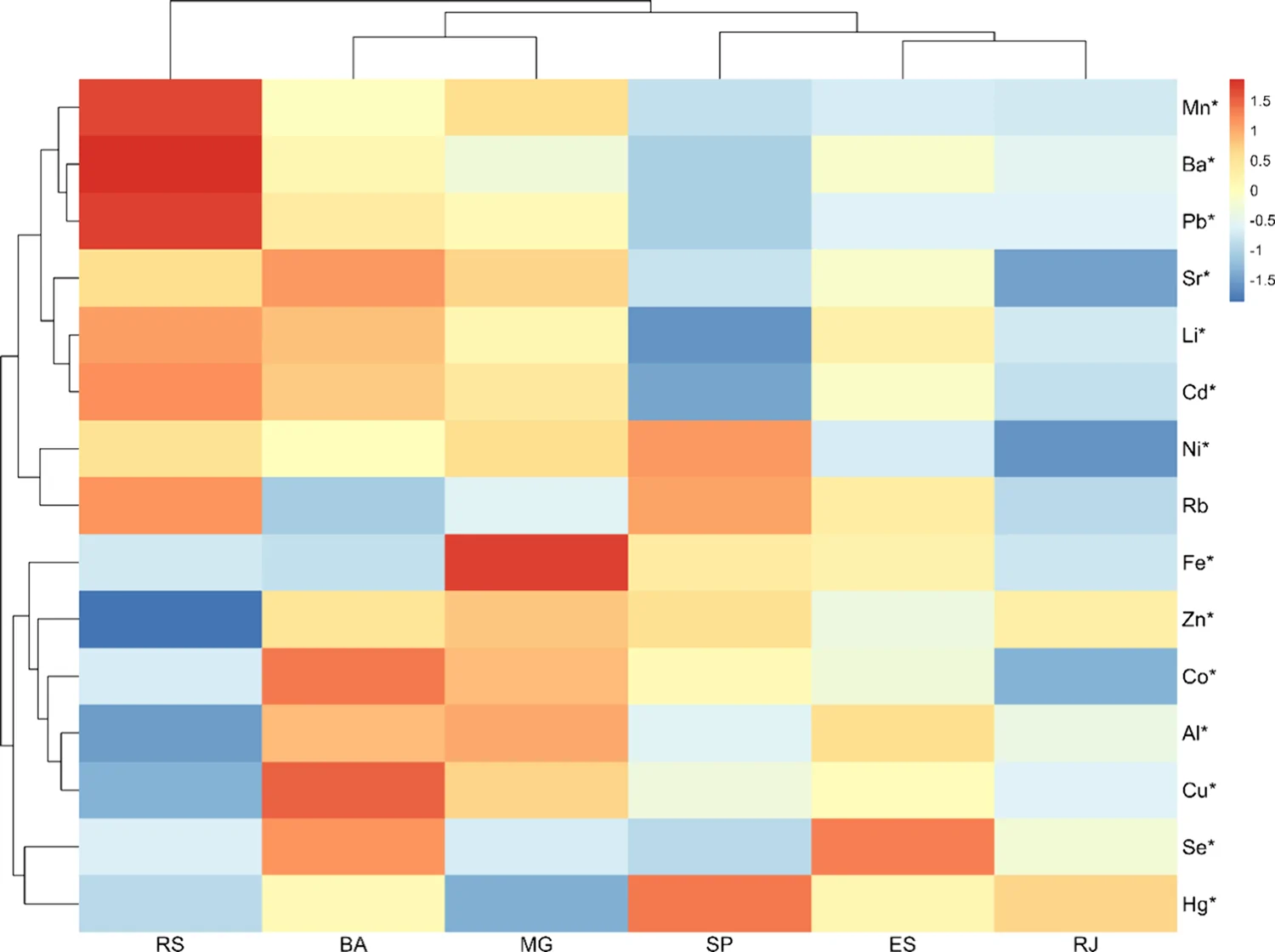
Hierarchical heatmap of region-specific metallomic fingerprints. Median standardized plasma metal concentrations (row-wise *z*-scores) are shown across Brazilian study centers, with hierarchical clustering applied to both metals and regions. An asterisk (*) indicates metals showing the strongest regional deviations (top absolute *z*-scores), highlighting key contributors to geographic differentiation in metallomic exposure profiles.

More specifically, hierarchical clustering identified a prominent Rb–Mn–Pb cluster strongly associated with Rio Grande do Sul, consistent with the combined contribution of geochemical background, agricultural fungicide use, and industrial activities. Espírito Santo was characterized by a Se-dominant cluster, whereas Bahia showed stronger association with Sr and Cu. São Paulo, in turn, exhibited a Ni–Hg-oriented profile, likely reflecting the interaction between urban-industrial influences and dietary seafood intake. These clustered multimetal configurations reinforce that geographic differentiation emerges from coordinated elemental signatures rather than isolated metal effects.

### 3.4 Supervised Machine Learning and Geographic Exposomic Signatures (GExOS)

Unsupervised analyses revealed structured, yet overlapping, regional metallomic patterns, and we therefore next implemented supervised machine learning models to rigorously assess whether these signatures could reliably distinguish individuals by geographic origin. In this context, the observed metallomic profiles can be interpreted as components of an GExOS, reflecting the combined influence of environmental, dietary, and local geochemical determinants associated with geographic location (Figure 3).

**Figure 3:**
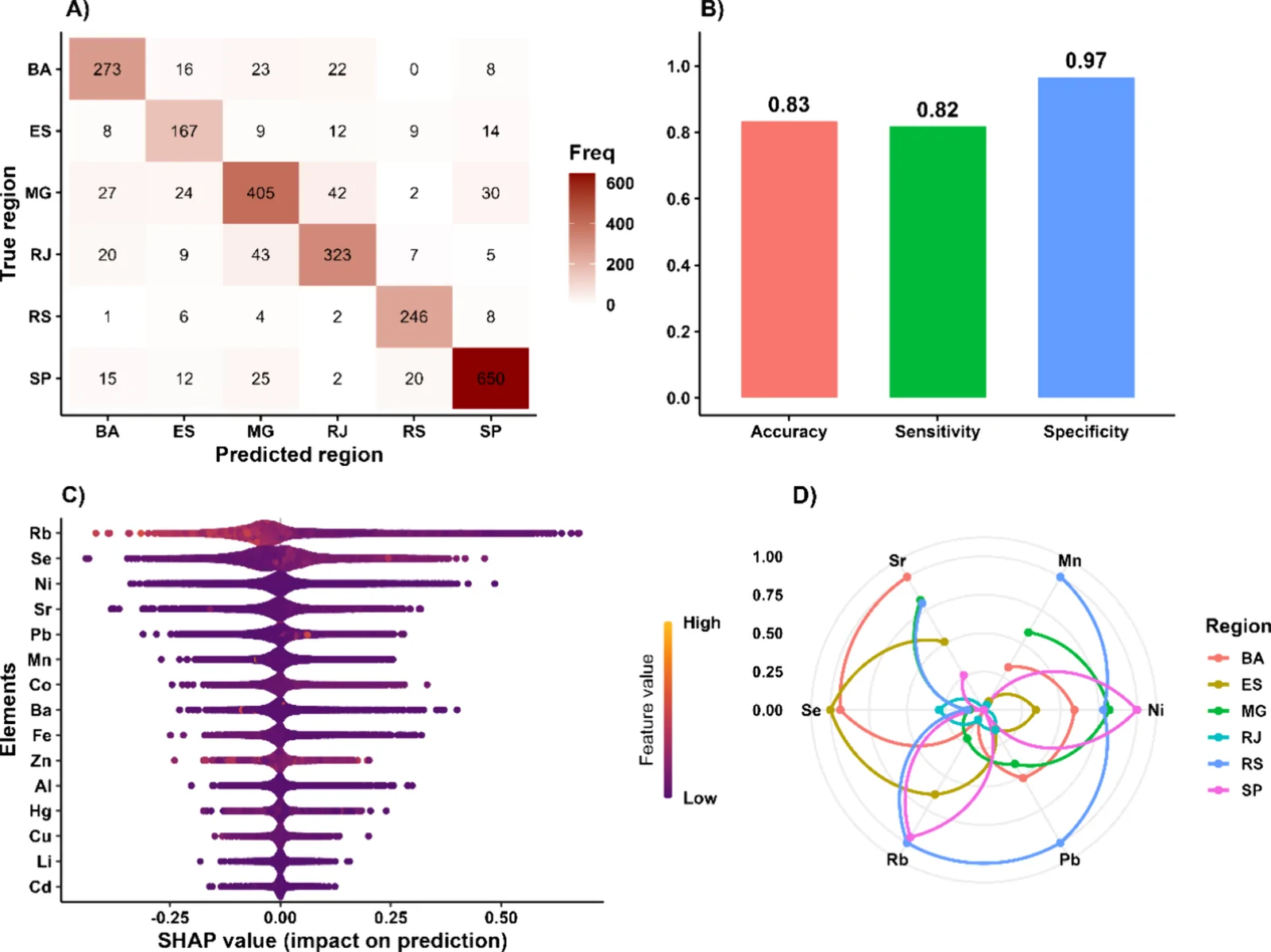
Supervised machine learning prediction of Brazilian regions based on plasma metallomic profiles using an XGBoost model. (A) Multiclass confusion matrix showing agreement between true and predicted regions across study centers. (B) Overall classification performance metrics, including accuracy, sensitivity, and specificity. (C) Global SHAP summary plot indicating relative contribution and directionality of individual metals in driving predictions across all individuals, with colors indicating low to high feature values. (D) Regional metallomic fingerprints based on the top SHAP-ranked metals, illustrating distinct multimetal exposure patterns characterizing the six Brazilian state capitals included in the study (BA, ES, MG, RJ, RS, and SP).

Another relevant contribution of this study is the exploratory assessment of plasma metal and metalloid profiles as potential markers for geographic differentiation. We developed an eXtreme Gradient Boosting (XGBoost) model to classify Brazilian adults based on the concentrations of 15 metals and metalloids. Region-specific patterns in plasma metal composition were identified, indicating that metallomic profiles vary according to geographic origin. These differences may partly reflect regional variability in nutritional habits and environmental exposures across Brazil.^22,24,25^

The multinomial classification model demonstrated satisfactory overall performance, achieving an accuracy of 0.83, sensitivity of 0.82, and specificity of 0.97 (Figure 3B). These results indicate that integrated metallomic profiles encode spatially structured information related to regional exposure patterns.

Beyond global metrics, the confusion matrix revealed clear region-specific classification behavior (Figure 3A). Rio Grande do Sul (Porto Alegre, Southern Brazil) and São Paulo (São Paulo, Southeastern Brazil) exhibited the most distinctive metallomic signatures, achieving the highest proportions of correct classification relative to their sample sizes (92.1 and 89.8%, respectively).

Although Minas Gerais (Belo Horizonte, Southeastern Brazil) accounted for a high absolute number of correct predictions, its proportional accuracy (76.4%) was comparable to those observed in Bahia (Salvador, Northeastern Brazil; 79.8%), Rio de Janeiro (Rio de Janeiro, Southeastern Brazil; 79.3%), and Espírito Santo (Vitória, Southeastern Brazil; 76.2%).

Higher misclassification among these latter regions suggest greater overlap in their metallomic profiles, potentially reflecting shared geographic, environmental, and anthropogenic characteristics across coastal and noncoastal areas of the Southeast and Northeast. Such similarities may reduce the discriminatory power of metallomic signatures between neighboring or environmentally comparable states.

Similar approaches have been reported in previous studies exploring the use of elemental profiles for geographic differentiation. For instance, the concentrations of 14 chemical elements (Cu, Zn, Co, Fe, Cr, Mn, Cd, As, Pb, Ni, P, Ca, K, and Mg) in hair samples were used to identify individuals from different regions of Kazakhstan.^28^ In that study, regional specificity in elemental levels was largely attributed to differences in the intensity and nature of local technogenic and environmental burdens.^28^

Using multivariate analytical approaches, Huang and Beauchemin examined elemental profiles in human hair samples (Li, Mo, S, Sr, Cr, K, Ni, Zn, and Pb) to infer individual’s ethnicity and gender, highlighting the potential forensic applications of metallomic data.^29^

These findings support the concept that multielemental signatures reflect region-specific exposure patterns. In forensic science, such profiles can support geographic attribution, human identification, and the reconstruction of exposure history, particularly when conventional identifiers are unavailable or limited.^28,29^ From a regulatory perspective, metallomic data can be applied to environmental monitoring, identification of populations at risk of elevated metal exposure, and evaluation of the effectiveness of public health interventions and environmental policies.^9,10,30^

Beyond traditional methodologies, the present study employs supervised machine learning to model the complex, multivariate relationships inherent in metallomic data sets.^31^ This strategy facilitates not only the identification of region-specific elemental signatures but also the robust classification of novel samples.^32^ It substantially enhances the applicability of metallomics in environmental surveillance, exposure mapping, population health risk stratification, and nutritional pattern inference, as well as in forensic and regulatory sciences. Collectively, these findings underscore the added value of supervised learning frameworks in improving discrimination accuracy and supporting objective, data-driven geographic attribution.

To elucidate the drivers of geographic classification, we assessed the relative importance of each variable using SHAP interpretability methods. Rb emerged as the most influential predictor, followed by Se, Ni, Sr, Pb, and Mn (Figure 3C). The broad distribution of SHAP values suggests substantial interindividual variability, consistent with heterogeneous exposure pathways within regions.

Finally, regional fingerprints constructed from the top SHAP-ranked metals illustrate that each Brazilian center is characterized by a distinct multimetal configuration rather than a single-element effect (Figure 3D). Rio Grande do Sul (RS) shows comparatively higher contributions from Rb, Mn, and Pb, whereas São Paulo (SP) are more strongly influenced by Ni, Espírito Santo (ES) by Se, and Bahia (BA) by Sr. These regional signatures are biologically plausible within the context of Brazil’s well-established dietary heterogeneity. Seafood-rich dietary patterns in coastal and metropolitan settings such as São Paulo and Rio de Janeiro may contribute to Hg and Ni variability, whereas cocoa-derived products may help explain the Se-enriched profile observed in Espírito Santo. In Rio Grande do Sul, higher consumption of rice, mate tea, wine, and coffee likely contributes to elevated systemic Rb levels.

In this context, the emergence of Rb as the primary geographic discriminator likely reflects the combined influence of regional dietary habits and soil geochemistry. Edaphic Rb concentrations are known to vary across Brazil, with higher levels reported in Rio Grande do Sul than in Minas Gerais,^33^ consistent with the plasma differences observed here (Figure 2). Because Rb occurs in staple foods and beverages such as rice, coffee, tea, and wine, regional variability in both soil availability and food consumption likely reinforces its value as an internal biomarker of geographic origin.^34,35^

Notably, studies incorporating Rb within human biomonitoring frameworks remain limited. In this context, our findings highlight the importance of including Rb in future human biomonitoring studies, given its potential to capture geographic variability in exposure and to improve the characterization of environmental and dietary determinants of metal profiles.

Importantly, the comparison between unsupervised and supervised approaches highlights their complementary roles in GExOS discovery. While PCA–UMAP and hierarchical clustering effectively revealed continuous regional gradients and latent multimetal structures, the supervised XGBoost framework transformed these subtle patterns into quantitatively predictive signatures with high discrimination performance. Moreover, SHAP-based interpretability enabled explicit identification of the metals driving regional differentiation, demonstrating the clear added value of supervised learning for translating latent exposure gradients into actionable and biologically interpretable geographic fingerprints.

Collectively, these results demonstrate that geographic discrimination is driven by coordinated multielement exposure signatures, supporting the existence of population-specific metallomic fingerprints shaped by complex environmental, dietary, and geochemical determinants.

### 3.5 Implications and Applications

We introduce an innovative analytical framework that integrates plasma metal and metalloid concentrations with supervised learning to classify adults by their Brazilian state of residence, revealing a distinctive chemical “digital fingerprint”. To the best of our knowledge, this is the first approach capable of distinguishing individuals from different Brazilian regions solely based on plasma elemental profiles. This methodology advances the concept of “GExOS” whereby geographically structured environmental exposures are reflected in internal chemical signatures measurable in human biological matrices.

The proposed GExOS framework has broad translational relevance. These signatures can be applied across multiple domains, including GExOS, human health and risk assessment, nutritional and dietary pattern inference, and forensic and regulatory sciences, highlighting the broad applicability of metallomics beyond descriptive biomonitoring (Figure 4).

**Figure 4:**
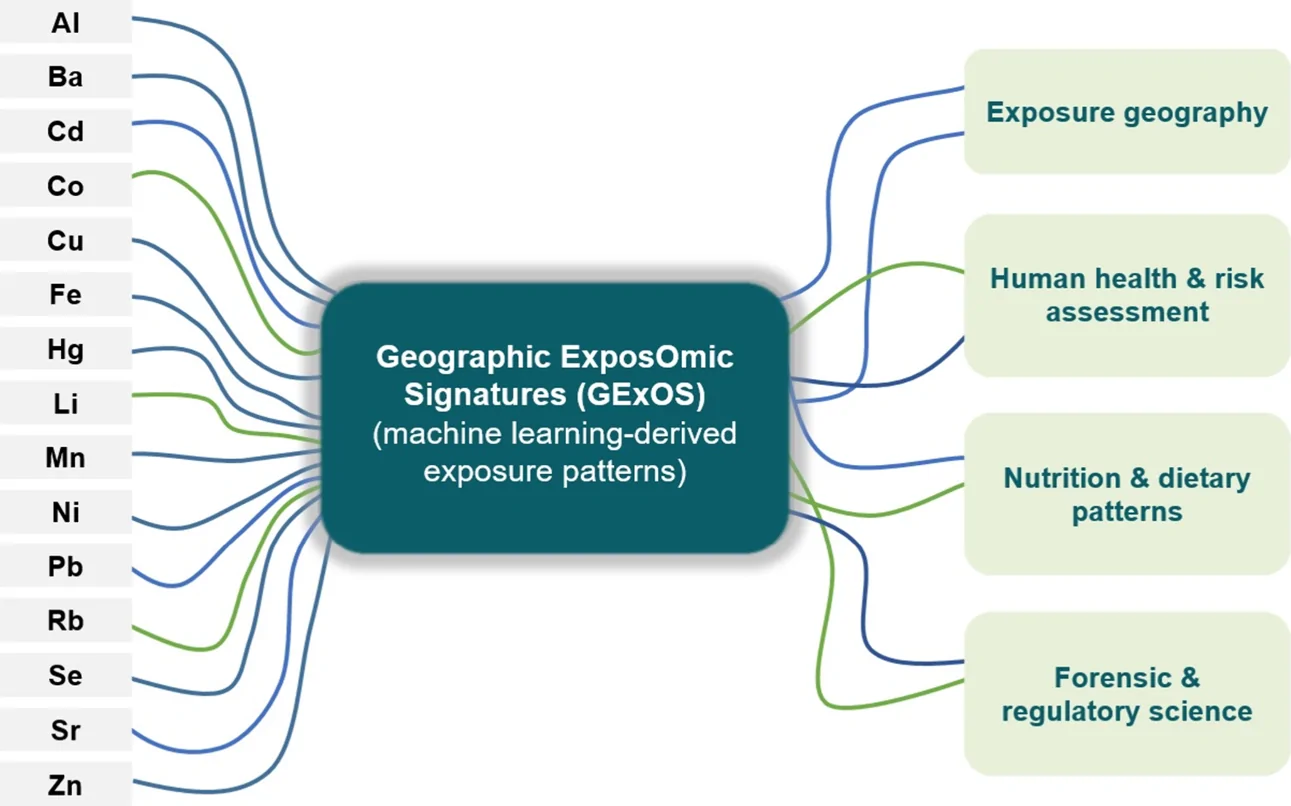
Conceptual framework illustrating the translational potential of machine learning-derived metallomic signatures.

Future integration of additional biological matrices, including urine and whole blood, will further strengthen this data set and enable a more comprehensive and multidimensional characterization of the Brazilian metallomic landscape.

### 3.6 Limitations

Several limitations should be acknowledged when interpreting these findings. First, the cross-sectional design does not allow assessment of temporal variation in exposure and precludes causal inference regarding the influence of geographic factors on metal bioaccumulation. In addition, plasma concentrations primarily reflect recent exposure (ranging from hours to weeks for most elements) and may therefore fail to capture longer-term or cumulative body burdens.^36^

Another limitation relates to the geographic granularity of the available data. Although state-capital level classification enabled robust identification of Geographic Exposomic Signatures, finer spatial resolution (e.g., neighborhood, municipality, or geocoded residential area) was not consistently available across all study centers. Future studies integrating GIS-derived environmental, urban, and food-system variables may substantially improve the spatial resolution and interpretability of GExOS.

Second, the ELSA-Brasil cohort is composed of civil servants from universities and research institutions in Brazilian state capitals, representing a relatively well-educated and economically stable segment of the Brazilian population. This characteristic may limit the generalizability of the findings to broader population groups, particularly those experiencing different occupational exposures or socioeconomic gradients in metal burden. Nevertheless, the internal validity of geographic comparisons is likely maintained, as similar recruitment procedures were applied across all study centers.

Third, plasma metal concentrations may be influenced by individual health conditions, medication use, and physiological states (e.g., inflammation, renal function, or pregnancy), which were not fully accounted for in the classification models.^30^

Finally, the machine learning models were developed and evaluated within a single cohort, using internal training and test sets. Replication in independent Brazilian samples from other states, as well as in cohorts from different countries, will be necessary to confirm the robustness, generalizability, and temporal stability of the identified metallomic fingerprints.

## 4 Conclusion

Taken together, these findings support metallomics, coupled with machine learning, as a practical and scalable strategy for operationalizing GExOS in human population studies. Using high-quality human biomonitoring data from the ELSA-Brasil cohort as proof of concept, we have shown that integrated metallomic profiles encode geographically structured exposure information, which can be translated into interpretable and predictive population-level signatures. Beyond methodological advances, this framework opens new perspectives for exposure-informed applications, including environmental surveillance and GExOS, population health risk stratification, nutritional pattern inference, and forensic and regulatory sciences, underscoring its broad translational relevance in environmental health research.

The cross-sectional design and the specific socioeconomic characteristics of the study population may limit causal inference and the generalizability of these findings. Future research should focus on longitudinal validation, external replication in diverse populations, and integration with additional environmental and biological data layers to advance its potential for precision public health applications.

## Supplementary Material



## Notes

Additional experimental details, including analytical quality assurance and quality control parameters for plasma trace element determination by ICP-MS and descriptive statistics of subject-specific plasma metal/metalloid levels by states (μg/L) (PDF)

## References

[1] Hasin Y., Seldin M., Lusis A. (2017). Multi-omics
approaches to disease. Genome biology.

[2] Machado I., Gambino D. (2024). Metallomics: Metallomics:
an essential tool for the
study of potential antiparasitic metallodrugs. ACS Omega.

[3] Haraguchi H. (2017). Metallomics:
the history over the last decade and a future outlook. Metallomics.

[4] Zhang Y., He J., Jin J., Ren C. (2022). Recent advances in the application
of metallomics in diagnosis and prognosis of human cancer. Metallomics.

[5] Ros A. C., Bacci S., Luna A., Legaz I. (2022). Forensic impact of
the omics science involved in the wound: a systematic review. Frontiers in medicine.

[6] Azuaje F., Devaux Y., Wagner D. (2009). Computational biology
for cardiovascular
biomarker discovery. Briefings in bioinformatics.

[7] Wang S., Chanock S., Tang D., Li Z., Edwards S., Jedrychowski W., Perera F. P. (2010). Effect of gene-environment
interactions
on mental development in African American, Dominican, and Caucasian
Mothers and Newborns. Annals of human genetics.

[8] Caberlotto L., Lauria M. (2015). Systems biology meets-omic
technologies: novel approaches
to biomarker discovery and companion diagnostic development. Expert Review of Molecular Diagnostics.

[9] Bocato M. Z., Ximenez J. P. B., Hoffmann C., Barbosa F. (2019). An overview of the
current progress, challenges, and prospects of human biomonitoring
and exposome studies. J. Toxicol. Environ. Health,
Part B.

[10] Leinardi R., Huaux F. (2025). Uncovering exposome-related
diseases through the pathologic metallome:
a novel approach for clinical populations. Frontiers
in Toxicology.

[11] Kim M. J., Heo M., Kim S. J., Song H. E., Lee H., Kim N.-E., Shin H., Do A. R., Kim J., Cho Y. M., Hong Y.-S., Kim W. J., Won S., Yoo H. J. (2024). Associations
between plasma metabolites and heavy metal exposure in residents of
environmentally polluted areas. Environ. Int..

[12] Matus P., Sepúlveda-Peñaloza A., Urquidi C. (2025). Geographical variations
in metal exposure and metabolic disorders in Chile using an exposome
approach. Sci. Rep..

[13] Tamayo-Ortiz M., Riojas-Rodríguez H., Téllez-Rojo M. M., Boischio A., Mañay N., Menezes-Filho J. A., Queirolo E. I., Cortés S., Kordas K. (2022). A call for biomonitoring
systems in Latin America and the Caribbean: considerations for potentially
toxic metals/metalloids. Annals of global health.

[14] Morabito A., De Simone G., Pastorelli R., Brunelli L., Ferrario M. (2025). Algorithms
and tools for data-driven omics integration to achieve multilayer
biological insights: a narrative review. Journal
of translational medicine.

[15] Arabshahi S., Davoodi Karsalari P., Baghsheikhi H., Mogharrabi F., Samieefar N., Rezaei N. (2026). The use of machine learning algorithms
in the identification of novel biomarkers for disorders of the immune
system. Advances in Biomarker Sciences and Technology.

[16] Aquino E. M. L., Barreto S. M., Benseñor I. M., Carvalho M. S., Chor D., Duncan B. B., Lotufo P. A., Mill J. G., Molina M. D. C., Mota E. L. A., Passos V. M. A., Schmidt M. I., Szklo M. (2012). Brazilian
Longitudinal Study of Adult Health (ELSA-Brasil): Objectives and Design. Am. J. Epidemiol..

[17] Benseñor I. M., Griep R. H., Pinto K. A., Faria C. P. D., Felisbino-Mendes M., Caetano E. I., Albuquerque L. S., Schmidt M. I. (2013). Routines of organization
of clinical tests and interviews in the ELSA-Brasil investigation
center. Revista de saude publica.

[18] Schmidt M. I., Duncan B. B., Mill J. G., Lotufo P. A., Chor D., Barreto S. M., Aquino E. M. L., Passos V. M. A., Matos S. M. A., Molina M. C. B., Carvalho M. S., Benseñor I. M. (2015). Cohort
Profile: Longitudinal Study of Adult Health (ELSA-Brasil). International journal of epidemiology.

[19] Lee P. H., Macfarlane D. J., Lam T. H., Stewart S. M. (2011). Validity of the
International Physical Activity Questionnaire Short Form (IPAQ-SF):
A Systematic Review. International journal of
behavioral nutrition and physical activity.

[20] World Health Organization . Obesity: Preventing and Managing the Global Epidemic; WHO Technical Report Series 894; WHO: Geneva, 2000. 11234459

[21] Corrêa L. G. (2024). Four concepts
to think from the South. Int. J. Cult. Stud..

[22] Lopes A., Ruiz R., Ribeiro R., Cantelmo W. (2023). linkages in the metal
mining industry: local job multipliers in Brazil. Resources Policy.

[23] Morais D. A., Sousa W. T., Salles G. P., Rufo C. M., Souza M. C. O., Domingo J. L., Lotufo P. A., Benseñor I., Barbosa F. (2026). Human biomonitoring of plasma trace
elements in Brazilian adults: distribution patterns and sociodemographic
determinants in the ELSA-Brasil cohort. Environmental
Research.

[24] Zanoteli E., Rocha J. C. C., Narumia L. K., Fireman M. A. T., Moura L. S., Oliveira A. S. B., Gabbai A. A., Fukuda Y., Kinoshita M., Toda T. (2002). Fukuyama-Type Congenital Muscular Dystrophy: A Case Report in the
Japanese Population Living in Brazil. Acta neurologica
scandinavica.

[25] Burgon V. H., Silva M. L. N., Milani R. F., Morgano M. A. (2024). Trace Elements in
Bean-to-Bar Chocolates from Brazil and Ecuador. Journal of Trace Elements in Medicine and Biology.

[26] Brum A. L., Baggio D. K., Kolosque F. P., Goi M. A. C. B. (2020). Tobacco production
chain in Southern Brazil: study of the composition and relationship
of its agents. Research, Society and Development.

[27] do
Nascimento S. N., Barth A., Göethel G., Baierle M., Charão M. F., Brucker N., Moro A. M., Bubols G. B., Sobreira J. S., Sauer E., Rocha R., Gioda A., Dias A. C., Salles J. F., Garcia S. C. (2015). Cognitive
deficits and ALA-D-inhibition in children exposed to multiple metals. Environmental Research.

[28] Mussabekova S. A., Mkhitaryan X. E. (2021). Elemental composition of hair as
a marker for forensic
human identification. Journal of Forensic and
Legal Medicine.

[29] Huang L., Beauchemin D. (2014). Ethnic background and gender identification
using electrothermal
vaporization coupled to inductively coupled plasma optical emission
spectrometry for forensic analysis of human hair. Journal of Analytical Atomic Spectrometry.

[30] Martinez-Morata I., Sobel M., Tellez-Plaza M., Navas-Acien A., Howe C. G., Sanchez T. R. (2023). A state-of-the-science
review on
metal biomarkers. Current environmental health
reports.

[31] Zitnik M., Nguyen F., Wang B., Leskovec J., Goldenberg A., Hoffman M. M. (2019). Machine learning for integrating data in biology and
medicine: Principles, practice, and opportunities. Information Fusion.

[32] Noorbakhsh J., Chandok H., Karuturi R. K. M., George J. (2019). Machine learning
in
biology and medicine. Advances in Molecular
Pathology.

[33] Pérez, D. V. ; Saldanha, M. F. D. C. ; Meneguelli, N. D. A. ; Moreira, J. C. ; Vaitsman, D. S. Geoquímica de alguns solos brasileiros. EMBRAPA-CNPS, 1997.

[34] Batista B. L., Souza V. C. O., da
Silva F. G., Barbosa F. (2010). Survey of 13 trace
elements of toxic and nutritional significance
in rice from Brazil and exposure assessment. Food Addit. Contam..

[35] Debastiani R., Amaral L., Dias J. F. (2018). Rubidium
in the elemental composition
of Brazilian coffee. International Journal of
PIXE.

[36] Coradduzza D., Sanna A., Di Lorenzo B., Congiargiu A., Marra S., Cossu M., Tedde A., De Miglio M. R., Zinellu A., Mangoni A. A., Aligio Cogoni A., Madonia M., Carru C., Medici S. (2025). Associations between
plasma and urinary heavy metal concentrations and the risk of prostate
cancer. Sci. Rep..

