# Supplementary material for: Machine Learning-Enabled Metallomics Reveals Geographic Exposomic Signatures in a Large Brazilian Cohort
Source: Environ Health (Wash). 2026 May 20;4(8):1843–52. doi: 10.1021/envhealth.6c00155 (PMC13504570; doi:10.1021/envhealth.6c00155)
Supplement: Supplementary file 1 [file eh6c00155_si_001.pdf]

Supporting information

Machine Learning–Enabled Metallomics Reveals Geographic Exposomic Signatures in a Large Brazilian Cohort

Déborah Araújo Morais [a], Wellington Tavares de Sousa Júnior [a], Gabriela Pereira de Salles [a], Marilia Cristina Oliveira Souza [b], Jose L. Domingo [c], Paulo Lotufo [d], Isabela M. Bensenor [d], Fernando Barbosa Jr [a*].

[a] Laboratório de Toxicologia Analítica e de Sistemas (ASTOx). Departamento de Análises Clínicas, Toxicológicas e Bromatológicas, Faculdade de Ciências Farmacêuticas de Ribeirão Preto, University of São Paulo, Ribeirão Preto, SP, Brazil.

[b] University of Sao Paulo, School of Pharmaceutical Sciences of Ribeirao Preto, Department of Biomolecular Sciences. Av. do Café s/n, 14040-903, Ribeirao Preto, Sao Paulo, Brazil.

[c] Universitat Rovira i Virgili, Laboratory of Toxicology and Environmental Health, School of Medicine, Sant Llorenç 21, 43201 Reus, Catalonia, Spain.

[d] Centro de Pesquisa Clínica e Epidemiológica, Hospital Universitário, University of São Paulo, São Paulo, SP, Brazil.

* Corresponding author.

E-mail addresses: fbarbosa@fcfrp.usp.br (F. Barbosa).

Table S1: Analytical quality assurance and quality control parameters for plasma trace element determination by ICP-MS. Based on the analysis of the reference material QM-S-Q1907 and spiked plasma samples.

Element	Detection frequency in samples (%)	LOD (µg/L)	Intra-day precision (%RSD)	Inter-day precision (%RSD)	Recovery (%)	Internal standard	Calibration range (µg/L)	Matrix-matched calibration	Correlation Coefficient (r)
Li*	100	0.0004	2.3	6.1	98-106	Rh	0.1-50	Yes	0.9999
Al	100	0.01	8.6	3.0	96-110	Rh	0.1-50	Yes	0.9994
Mn	100	0.007	2.2	2.2	106-110	Rh	0.1-50	Yes	0.9999
Fe*	100	2.0	5.5	6.4	101-112	Rh	0.1-100	Yes	0.9975
Co	100	0.006	3.4	1.4	104-114	Rh	0.1-50	Yes	0.9999
Ni	100	0.01	2.8	4.8	97-110	Rh	0.1-50	Yes	0.9999
Zn	100	0.32	4.0	3.5	98-107	Rh	0.1-100	Yes	0.9980
Cu	100	0.03	2.8	5.9	95-104	Rh	0.1-50	Yes	0.9994
Se	100	0.01	1.1	2.6	95-99	Rh	0.1-50	Yes	0.9999
Rb*	100	0.004	4.0	4.4	96-99	Rh	0.1-50	Yes	0.9999
Sr*	100	0.008	2.6	1.3	99-101	Rh	0.1-50	Yes	0.9999
Cd	100	0.003	7.0	4.1	99-109	Rh	0.1-50	Yes	0.9999
Ba	100	0.001	3.3	6.4	92-99	Rh	0.1-50	Yes	0.9999
Hg	99.5	0.004	4.7	6.3	97-110	Rh	0.1-5	Yes	0.9999
Pb	100	0.004	4.2	4.5	96-105	Rh	0.1-50	Yes	0.9999

*spiked samples

Table S2: Descriptive statistics of subject-specific plasma metal/metalloid levels by states (µg/L)

Metal	Bahia median (IQR)	Minas Gerais median (IQR)	Espírito Santo median (IQR)	Rio de Janeiro median (IQR)	São Paulo median (IQR)	Rio Grande do Sul median (IQR)
Li	1.0 (0.79 - 1.3)	0.98 (0.66 - 1.4) [a]	0.99 (0.71 - 1.2) [a]	0.90 (0.73 - 1.1) [a, b]	0.82 (0.65 - 1.0) [a, b, c, d]	1.1 (0.70 - 1.4) [c, d, e]
Al	24.1 (17.0 - 34.3)	24.4 (17.2 - 37.5)	23.5 (17.8 - 32.6)	21.0 (16.5 - 28.4) [a, b, c]	20.6 (15.9 - 26.8) [a, b, c]	18.3 (13.9 - 24.3) [a, b, c, d, e]
Mn	3.4 (2.8 - 4.6)	3.7 (2.9 - 5.5) [a]	3.1 (2.6 - 3.8) [a, b]	3.0 (2.6 - 3.6) [a, b, c]	3.0 (2.0 - 4.3) [a, b, c]	4.2 (3.0 - 7.6) [a, b, c, d, e]
Fe	1469 (1183 - 1791)	1730 (1431 - 2088) [a]	1580 (1277 - 1954) [a, b]	1479 (1229 - 1756) [b, c]	1593 (1263 - 1951) [a, b, d]	1480 (1186 - 1832) [b, c, e]
Co	0.60 (0.51 - 0.70)	0.57 (0.48 - 0.73) [a]	0.51 (0.44 - 0.62) [a, b]	0.45 (0.38 - 0.56) [a, b, c]	0.53 (0.44 - 0.62) [a, b, d]	0.48 (0.40 - 0.57) [a, b, c, d, e]
Ni	3.9 (3.0 - 4.8)	4.4 (3.3 - 6.0) [a]	3.4 (2.9 - 3.9) [a, b]	2.7 (2.0 - 3.6) [a, b, c]	4.7 (3.9 - 6.6) [a, b, c, d]	4.3 (3.5 - 5.1) [a, c, d, e]
Zn	1025 (894 - 1150)	1050 (903 - 1290) [a]	953 (853 - 1084) [a, b]	1009 (873 - 1312) [b, c]	1030 (828 - 1371) [b, c]	827 (733 - 920) [a, b, c, d, e]
Cu	985 (835 – 1153)	963 (826 – 1117)	947 (817 – 1114)	930 (803 – 1069) [a, b]	937 (794 – 1103) [a, b]	911 (754 – 1084) [a, b, c, e]
Se	98 (84.2 - 114)	75 (63 - 92) [a]	100 (88.9 - 116) [a, b]	80 (68 - 96) [a, b, c]	72 (62 - 88) [a, b, c, d]	75 (61 - 95) [a, c, d, e]
Rb	207 (161 - 315)	244 (188 - 304) [a]	316 (271 - 363) [a, b]	220 (170 - 258) [a, b, c]	371 (322 - 426) [a, b, c, d]	378 (323 - 435) [a, b, c, d]
Sr	54 (37.6 - 81)	50 (34.7 - 103)	43.1 (35.7 - 55) [a, b]	31.0 (23.6 - 41.8) [a, b, c]	37.2 (29.0 - 47.0) [a, b, c, d]	49.9 (32.4 - 110) [a, c, d, e]
Cd	0.084 (0.059 - 0.12)	0.079 (0.050 - 0.14) [a]	0.070 (0.049 - 0.11) [a, b]	0.058 (0.042 - 0.084) [a, b, c]	0.049 (0.035 - 0.069) [a, b, c, d]	0.092 (0.064 - 0.13) [b, c, d, e]
Ba	12.9 (10.1 - 18.9)	11.3 (6.0 - 18.0) [a]	11.8 (9.6 - 15.8) [b]	10.4 (8.0 - 14.3) [a, c]	8.5 (6.0 - 11.7) [a, b, c, d]	19.3 (14.2 - 24.9) [a, b, c, d, e]
Hg	0.45 (0.25 - 0.76)	0.30 (0.17 - 0.52) [a]	0.46 (0.26 - 0.77) [b]	0.51 (0.30 - 0.78) [a, b]	0.58 (0.32 - 0.97) [a, b, c, d]	0.35 (0.19 - 0.63) [a, b, c, d, e]
Pb	1.7 (1.26 - 2.73)	1.5 (0.98 - 2.8) [a]	1.2 (0.89 - 1.9) [a, b]	1.2 (0.81 - 2.1) [a, b]	0.97 (0.64 - 1.8) [a, b, c, d]	2.4 (1.6 - 3.4) [a, b, c, d, e]

A significance level of 5% (*p*-value ≤ 0.05), obtained through the Kruskal Wallis, followed by a post hoc Bonferroni test, between states median values: a = versus Bahia; b = versus Minas Gerais; c = versus Espírito Santo; d = versus Rio de Janeiro; e = versus São Paulo.
